# Pursuing More Aggressive Timelines in the Surgical Treatment of Traumatic Spinal Cord Injury (TSCI): A Retrospective Cohort Study with Subgroup Analysis

**DOI:** 10.3390/jcm10245977

**Published:** 2021-12-20

**Authors:** Tobias Bock, Raban Arved Heller, Patrick Haubruck, Tim Friedrich Raven, Maximilian Pilz, Arash Moghaddam, Bahram Biglari

**Affiliations:** 1 Heidelberg Trauma Research Group, Centre for Orthopaedics, Trauma Surgery and Paraplegiology, Heidelberg University Hospital, 69118 Heidelberg, Germany; raban.heller@med.uni-heidelberg.de (R.A.H.); patrick.haubruck@sydney.edu.au (P.H.); 2 Department of Anesthesiology and Intensive Care, Leipzig University Hospital, Liebigstraße 20, 04103 Leipzig, Germany; 3 Bundeswehr Hospital Berlin, Department of Traumatology and Orthopaedics, Septic and Reconstructive Surgery, 10115 Berlin, Germany; 4 Department of General Practice and Health Services Research, Heidelberg University Hospital, 69120 Heidelberg, Germany; 5 Raymond Purves Bone and Joint Research Laboratory, Kolling Institute, Institute of Bone and Joint Research, University of Sydney, St. Leonards, NSW 2006, Australia; 6 Department of Trauma, Hand and Reconstructive Surgery, University Hospital Goethe University, 60590 Frankfurt am Main, Germany; tim.raven@kgu.de; 7 Department of Medical Biometry, Institute of Medical Biometry and Informatics (IMBI), Heidelberg University Hospital, 69120 Heidelberg, Germany; pilz@imbi.uni-heidelberg.de; 8 PrivatÄrztliches Zentrum Aschaffenburg, 63739 Aschaffenburg, Germany; info@profmoghaddam.com; 9 Department of Paraplegiology, BG Trauma Centre Ludwigshafen, Ludwig-Guttmann-Straße 13, 67071 Ludwigshafen, Germany

**Keywords:** traumatic spinal cord injury, timing, decompressive surgery, laminectomy, neurological recovery, neurological outcome, AIS, biomarker

## Abstract

Background: The optimal timing of surgical therapy for traumatic spinal cord injury (TSCI) remains unclear. The purpose of this study is to evaluate the impact of “ultra-early” (<4 h) versus “early” (4–24 h) time from injury to surgery in terms of the likelihood of neurologic recovery. Methods: The effect of surgery on neurological recovery was investigated by comparing the assessed initial and final values of the American Spinal Injury Association (ASIA) Impairment Scale (AIS). A post hoc analysis was performed to gain insight into different subgroup regeneration behaviors concerning neurological injury levels. Results: Datasets from 69 cases with traumatic spinal cord injury were analyzed. Overall, 19/46 (41.3%) patients of the “ultra-early” cohort saw neurological recovery compared to 5/23 (21.7%) patients from the “early” cohort (*p* = 0.112). The subgroup analysis revealed differences based on the neurological level of injury (NLI) of a patient. An optimal cutpoint for patients with a cervical lesion was estimated at 234 min. Regarding the prediction of neurological improvement, sensitivity was 90.9% with a specificity of 68.4%, resulting in an AUC (area under the curve) of 84.2%. In thoracically and lumbar injured cases, the estimate was lower, ranging from 284 (thoracic) to 245 min (lumbar) with an AUC of 51.6% and 54.3%. Conclusions: Treatment within 24 h after TSCI is associated with neurological recovery. Our hypothesis that intervention within 4 h is related to an improvement in the neurological outcome was not confirmed in our collective. In a clinical context, this suggests that after TSCI there is a time frame to get the right patient to the right hospital according to advanced trauma life support (ATLS) guidelines.

## 1. Introduction

Traumatic spinal cord injury (TSCI) is considered one of the most severe injuries in traumatology. It often affects a predominantly young collective of patients [1,2] and requires experience and knowledge to ensure the best possible outcome. The physical and psychosocial [3] consequences for patients are severe and the resulting challenges affect not only the patient, but also society, and healthcare institutions likewise [4]. Worldwide, differing incidence (up to 246.0 per million) and prevalence (up to 1298 per million) data according to geography have been reported [1].

The injury affecting the spinal cord during TSCI is classified by primary and secondary damage [5]. While direct physical force induces the primary phase during trauma, the secondary phase is caused by pathological processes during the first hours and lasting up to several weeks post trauma [6]. Primary damage cannot be prevented, and therefore the containment of secondary damage remains crucial to improve therapy. Medical treatment options are currently focused on surgical procedures and standardized rehabilitation to prevent further damage and achieve the best possible neurological outcome. Addressing this problem, in the literature a variety of different approaches can be found. The current method of choice is surgical decompression to maximize the patient’s chances for neurological remission [7].

To date, the proper timing of surgical therapy is heavily discussed in the literature [8,9,10,11,12,13,14,15]. Despite several studies reporting limited benefits for patients [16,17,18,19,20], and even worsening damage through iatrogenic intervention [21], there is growing evidence that decompressing the spinal cord early after an injury has a clinical benefit [8,10,12,13,14,15,22,23,24,25,26]. In the most extensive prospective analysis to date on decompression in cervical TSCI including 313 cases, Fehlings et al. found evidence that surgery within 24 h of trauma is associated with a two-stage improvement in the American Spinal Injury Association (ASIA) Impairment Scale (AIS) [8]. Dvorak et al. provided corroborating evidence in a study with 470 patients from the Rick Hansen Spinal Cord Injury Registry [27] by showing that surgery performed within 24 h from injury was associated with higher odds for motor neurological recovery on the cervical, thoracic, or thoracolumbar level [13]. La Rosa et al. showed in a meta-analysis that “early” surgery (<24 h) led to a better neurological outcome than late (≥24 h) or conservative treatment [10].

Based on the results of our prior work in this field [9] and biological rationale, there is an incentive to examine the optimal timing of spinal cord-relieving surgery in the very first hours of TSCI. 

This study investigates the hypothesis “Surgical decompression 4 h after trauma has a beneficial effect on the neurological recovery of patients with TSCI”. Furthermore, we aimed to disclose the time frame in subgroups in which optimal surgical care should be provided. 

## 2. Materials and Methods

### 2.1. Study Design and Preconditions

This is a retrospective, monocentric cohort study. Data on predictor and outcome variables were collected prospectively as part of a research project investigating cytokine expression patterns in the post-traumatic course. Based on the International Spinal Cord Injury Core Data Set [28], descriptive data were obtained as well.

The local Ethics Committee approved the current study of the University of Heidelberg, Germany (S514/2011). It was registered 23.03.2016 (Study-ID: DRKS00009917, Universal Trial Number (UTN): U1111-1179-1620) at the German Clinical Trial Register (DRKS, Deutsches Register Klinischer Studien). All study participants or their authorized relatives signed an informed consent form and a voluntary participation agreement. They were informed that they could voluntarily decide to leave the study without negative consequences at any time.

Data collection and processing were performed according to good scientific practice. The manuscript was composed according to the STROBE (Strengthening the reporting of observational studies in epidemiology) statement [29]. The study was performed following the declaration of Helsinki [30]. 

### 2.2. Study Population: Inclusion and Exclusion Criteria

All patients suffering from TSCI with at least one spinal fracture accompanied by spinal cord compression were eligible for study participation. Validation was achieved by X-ray, computed tomography (CT), and magnetic resonance imaging (MRI). Yet, the management of life-threatening injuries took the highest priority and led to a delay in the surgical treatment of the spine. Cases with corresponding constellations were not considered.

**Figure 1:**
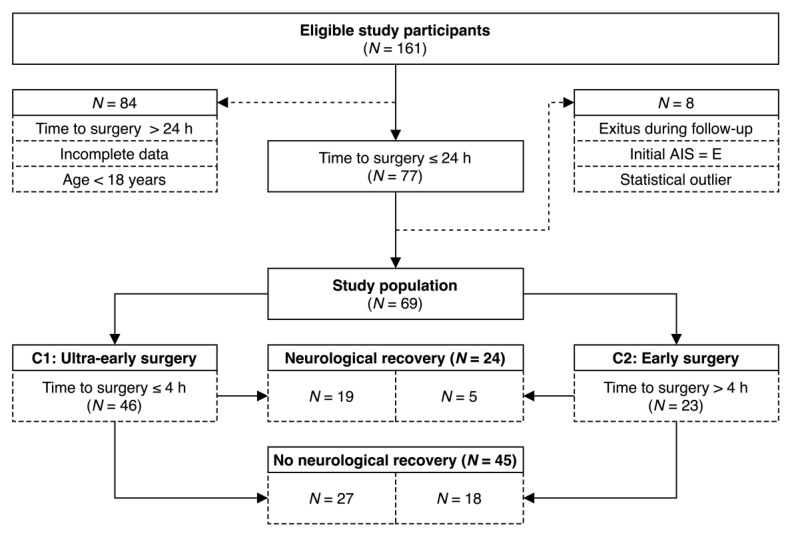
Flow diagram of the formation of the study population.

A total of 161 eligible study participants were admitted to the BG Trauma Centre Ludwigshafen due to a TSCI between 2012 and 2018. Figure 1 visualizes patient allocation and distribution of group sizes throughout the study.

In 84 cases, the following criteria led to exclusion: surgical treatment of the spinal cord after more than 24 h post injury, incomplete datasets, and age under 18 years. Out of the remaining 77 patients, 4 patients were excluded due to an initial AIS score E or exitus letalis in the follow-up period. We further performed an exclusion of outliers prior to the statistical analysis. For this purpose, we used Tukey fences with a factor of 3 [31]. Four records were removed accordingly (Supplementary Table S1). The remaining 69 patients formed the study population. With respect to our 2016 work [9], we were able to include approximately 35% more patients.

### 2.3. Grouping

This cutoff was chosen based on the results of our 2016 work and reflects the authors’ experience with the average time from accident to surgery in BG Ludwigshafen [9]. Patients were assigned to Cohort 1 (C1) or Cohort 2 (C2) (Figure 1). C1 (N = 46) includes all patients who experienced an intervention within 4 h after the injury. Participants treated between 4 and 24 h after the accident were summarized to C2 (N = 23). Prehospital care including transport as well as intrahospital care after admission (diagnostics, medical measures, and preparations for surgery) essentially determined the time from the accident to the operation.

### 2.4. Standardized Treatment

All included patients (N = 69) were hospitalized and underwent “early surgical treatment of the spinal cord” at BG Trauma Center Ludwigshafen, Germany, which consists of decompressive surgery and stabilization of the spine. Upon arrival, all patients received both anteroposterior and lateral radiographs and imaging using CT. Dorsal stabilization with decompression of the spinal cord was carried out in all study participants no later than 24 h after trauma. High-grade fractures were treated with open surgery and if needed the fractured vertebral body was replaced. In critically injured patients that required both dorsal and ventral stabilization of the spine during the initial surgery, the spinal cord was decompressed and dorsally stabilized while ventral stabilization was performed in a second surgery during the following 14 days. No further decompression measures were performed during the second, ventral surgery. During the follow up-period, no complications related to surgical procedures on the spinal cord were recorded. Participants were not treated with vasopressors, methylprednisolone sodium succinate, or similar corticoids during study participation. All patients received standardized physiotherapy and ergotherapy.

### 2.5. Outcome

The presence or absence of neurological recovery was the primary endpoint of the current study. According to the International Standards for Neurological Classification of Spinal Cord Injury (ISNCSCI; see Supplementary Table S2), AIS grades were determined to classify the neurological impairment [32,33]. Initial examinations (AIS initial) were performed within 72 h after admission in fully awake and responsive patients, as discussed by Fawcett et al. [34]. Final Assessments (AIS final) took place after a three-month follow-up period. Each ISNCSCI examination was performed by the head physical therapist with no blinding to the patient’s records. By comparing AIS initial and AIS final, the primary endpoint could be determined in terms of a positive AIS score conversion, which we equated with neurological recovery.

### 2.6. Variables of Interest

Necessary information such as gender, age at trauma, etiology of injury, type of plegia, neurological level of injury (NLI), and AO classification of the spinal fracture was collected. NLI was defined as the lowest neurological level in which both motor and sensory functions were intact.

The time from injury to “early surgical treatment of the spinal cord” was calculated based on the time stamps taken from the respective emergency-service logs and the Hospital Information System (HIS). While the actual time of the injury could not be validated, in general the emergency services in Germany arrive in a matter of minutes after injury. We utilized the time when emergency services were called as a substitute for the time of injury. Time from injury to admission, time from admission to “early surgical treatment of the spinal cord”, and surgery duration were determined as well.

### 2.7. Statistical Methods

We performed a statistical analysis of our prospectively collected data to determine significant differences between C1 and C2 concerning AIS-grade improvement. A post hoc analysis was utilized to determine the optimal point in time for surgical intervention, according to the Youden Index [35].

Testing for normality with Shapiro–Wilk was highly significant (W = 0.90111, *p*-value < 0.001). We therefore used nonparametric tests to assess the desired variables. The Kruskal–Wallis rank-sum test was used to compare nonparametric scaled variables. If categorical data was available as a 2 × 2 contingency table, they were analyzed with Boschloo’s test [36], otherwise, Fisher’s exact test for count data was used. All *p*-values quoted are to be interpreted descriptively as they were not adjusted for multiple testing as this is an exploratory post hoc analysis. All statistical tests used an α-level of 0.05, and statistical significance was defined as *p* > 0.05 (n.s), *p* < 0.05 (*), *p* < 0.01 (**), *p* < 0.001 (***). Univariate logistic regression was utilized to assess the predictive potential of the variables with respect to the criterion of neurological remission. The primary measure for the logistic regression model’s predictive performance was the area under the curve (AUC) of the Receiver-Operating-Characteristics (ROC) analysis [37]. All statistical calculations were performed with R studio version 1.3.1093 (R 4.0.2), applying the packages “fs”, “readxl”, “writexl”, “tidyr”, “dplyr”, “knitr”, “tidyverse”, “arsenal”, “gridtext”, “ggtext”, “glue” and “cutpointr”. Figures were created using the package “ggplot2” [38,39,40,41,42,43,44,45,46,47,48,49].

### 2.8. Power

Concerning power analysis in published works on the subject of timing in traumatic spinal cord injury, we found no evidence which could be used to make an educated guess. Rather, the field of power analysis in this area is fraught with problems. The heterogeneity of injury patterns strongly influences the calculation of the study population in the context of TSCI. The required number of cases to achieve significant power is high. A comparatively low incidence of this trauma also complicates the acquisition of a large number of cases.

### 2.9. Missing Data

From our data, 0 out of 77 records were removed due to missing values.

## 3. Results

### 3.1. Demographics

A total of 69 patients (57 male and 12 female) after traumatic spinal cord injury were observed over a period of three months. The mean age of the overall study population was 45.4 (*SD* = 18.2) years and ranged between 20.0 and 86.0 years. 

The two cohorts did not differ significantly in the neurological level of injury, *p* = 0.528. Cervical NLI was counted in 30 (43.5%) patients, thoracic NLI in 27 (39.1%), lumbar NLI in 12 (17.4%). There were no sacral cases. The classification of fractures was carried out according to the AO classification [50] and showed no significant differences. Fractures classified as type A were recorded in 35 (50.7%) cases, type B fractures in 13 (18.8%) cases, type C fractures in 21 (30.4%) cases (Table 1).

Furthermore, there were no significant differences between the two cohorts in terms of gender distribution or etiology. However, there was a difference in the average age distribution between C1 (*M* = 41.1, *SD* = 15.7) and C2 (*M* = 53.9, *SD* = 20.2), *p* = 0.010. A complete overview is given in Table 1.

### 3.2. Timing Characteristics

Overall, patients showed a mean time from injury to surgery of 229.4 (±72.7) min. Cohort 1 was treated on average within 191.7 (±35.6) min, Cohort 2 within 304.9 (±69.3) min.

**Figure 2:**
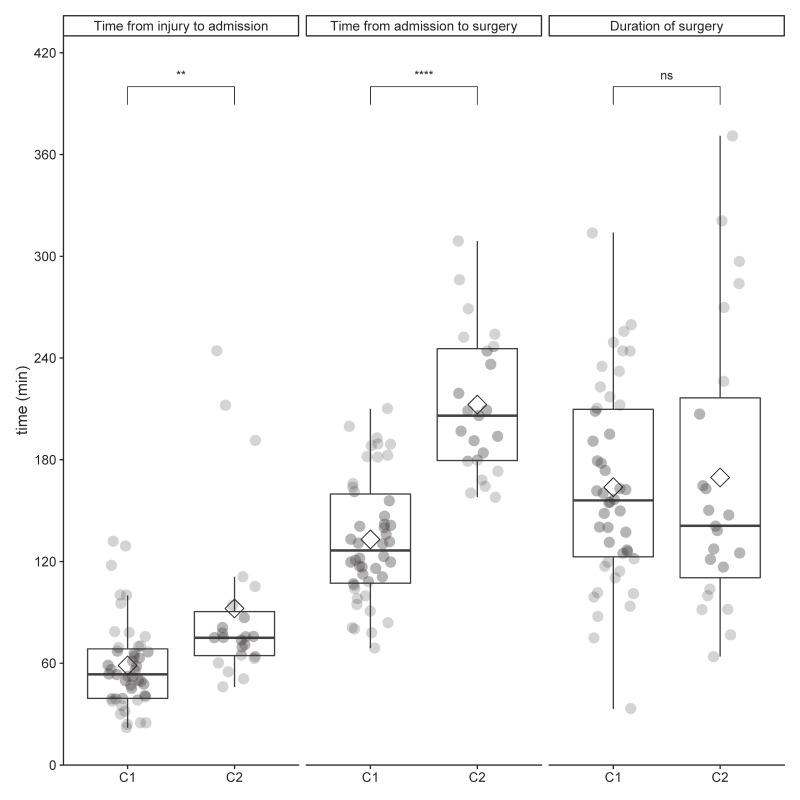
Overview of timing intervals of C1 vs. C2. *Note.* C1, Cohort 1: time from injury to injury <4 h; C2, Cohort 2: time from injury to injury = 4–24 h; Circles: raw data; Rhombus: mean; ns, not significant, *p* > 0.05; **, *p* < 0.01; ****, *p* < 0.0001.

C1 and C2 differed significantly in time from injury to admission (*p* < 0.001) and admission to surgery (*p* < 0.001). No significant differences could be seen in the duration of surgery. A comparison of the timing characteristics of C1 and C2 is shown in Figure 2.

A detailed overview of timing characteristics can be seen in Table 1.

### 3.3. Neurological Recovery

Neurological recovery was seen in 24/69 (34.8%) patients of the study population. In patients with a surgical treatment within 4 h, 19/46 (41.3%) patients had an improved neurological outcome while the remaining 27 (58.7%) showed no change in AIS grades. In contrast to this, 5/23 (21.7%) patients operated within 4 and 24 h showed an improved neurological outcome, while 18 (78.3%) patients did not. There was no significant difference to be found regarding neurological recovery between Cohort 1 and 2 (*p* = 0.112).

### 3.4. *Post hoc* Analysis

A post hoc analysis was performed to determine an optimal cutpoint and investigate subgroups regarding the primary outcome. 

#### 3.4.1. Cutpoint of Time from Injury to Surgery vs. Optimal Cutpoint

The model predicting the presence of neurological recovery indicated an AUC of 65.8% with a sensitivity of 79.2% and a specificity of 40.0%, given the predefined cutpoint of 4 h (240 min) from injury to surgery. Based on a minimum sensitivity of 85% and a maximum specificity, the data indicated an optimal cutpoint of 245 min. This cutpoint was associated with a sensitivity of 87.5% and a specificity of 37.8%.

#### 3.4.2. Subgroup Analysis

Following the indications of Wilson et al., the NLI subgroups were analyzed [22]. We focused on subgroups in terms of the neurological level of injury and the severity of TSCI. 

Regarding the neurological level of injury, a comparison showed that patients with thoracic NLI tended to have the worst outcome. Only 22.2% improved at least one step on AIS, followed by cervical spine injuries with 36.7%. The majority of lumbar cases showed an improvement of 58.3% (Table 2).

The cervical, thoracic, and lumbar groups tended to differ in their distribution of severity of TSCI (*p* = 0.053). While thoracic injuries mostly counted complete TSCI (70.4%), incomplete TSCI mostly appeared in the cervical (60.0%) and lumbar cases (58.3%).

**Figure 3:**
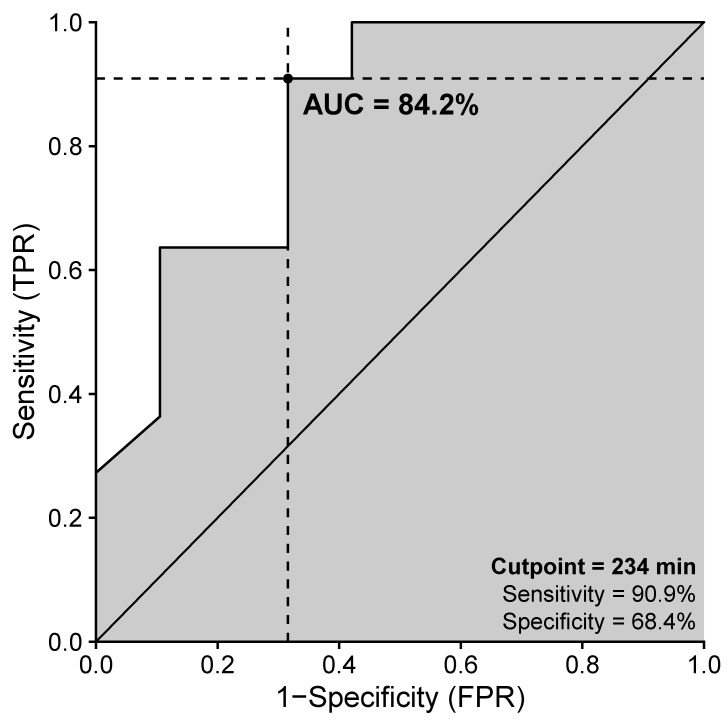
ROC curve of the optimal cutpoint for cervically injured patients. *Note.* The figure displays the respective optimal cutpoint by maximizing specificity for cervical TSCI, while sensitivity is set to a minimum of 85.0%. AUC, area under the curve; FPR, false positive rate; TPR, true positive rate.

Based on sensitivity over 85% and maximized specificity, the optimal cutpoint was estimated at 234 min after injury in cervically injured patients. The sensitivity was comparably high at 90.9%. The specificity was 68.4% and the AUC 84.2% (Figure 3).

In thoracically and lumbar injured cases, the estimate was lower, ranging from 284 (thoracic) to 245 min (lumbar) with an AUC of 51.6% and 54.3%.

Patients with incomplete TSCI presented higher odds of neurological recovery than patients with complete TSCI (51.5% vs. 19.4%, *p* = 0.006) (Table 3).

## 4. Discussion

Early timing (<24 h) of decompressive surgery is essential to achieve the best possible neurological outcome in the treatment of TSCI. Existing pathophysiological findings suggest that the time frame should be kept as short as possible. Thus, the exact time to surgically treat TSCI providing the best possible outcome remains unknown.

Large, prospective trials such as the heavily anticipated SCI-POEM study [51] remain scarce. Little evidence of the impact of surgery within 8 h on the neurological outcome of TSCI can be found, while available data show heterogeneous results. To our knowledge, surgical decompression within 4 h has not been investigated yet.

The data of the 2015 study conducted by Jug et al. showed a significant difference in favor of patients treated within 8 h after a 6-month follow-up [52]. In 2016, Grassner et al. suggested that intervening earlier than 8 h might increase the likelihood of functional improvement, as they observed significantly higher Spinal Cord Independence Measure (SCIM) scores in patients who underwent decompression within 8 h of injury [23].

Mattiassich et al. compared the neurological results between two cohorts with a “very-early” threshold (5 h) of cervically injured patients in a small (N = 49), retrospective, multicenter cohort study (ASCIS, Austrian Spinal Cord Injury Study) in 2017. Against their initial assumption, they saw a higher proportion of neurological recovery in the group treated after 5 h [12]. 

Compared to these previous studies, this study sought to determine whether surgical treatment of the spinal cord within 4 h affects neurological recovery in patients with TSCI within 3 months. We recruited 69 patients in a monocentric, retrospective cohort study based on prospectively acquired data, and measured their AIS improvement in a follow-up period of 3 months. 

In the current study, we observed 41.3% of patients had an improved neurological outcome in Cohort 1 compared to 21.7% in Cohort 2. Although these differences were at a nonsignificant level, this supports the conclusion that a surgical intervention within 8 h is not only feasible but has a positive influence on neurological recovery. The exact timing remained inconclusive, given the present data. Findings from the current study are supported by the results from our previous analysis [9], which showed no significant differences for neurological improvement in cohorts within “early” or “late” surgery.

### 4.1. *Post hoc* Analysis

#### 4.1.1. Optimal Cutpoint

Based on the available data, we were able to specify the optimal cutpoint of 245 min (4.08 h) after injury with an associated sensitivity of 87.5%. 

#### 4.1.2. Subgroups

Evaluating subgroups is highly demanded by many authors, as there is a lack of evidence in this field [22]. Therefore, we widened our post hoc analysis to subgroups of NLI and the severity of TSCI as they are reported to predict neurological recovery [53].

Our data showed that patients with lumbar TSCI tended to recover better than those with the cervical type, while thoracic TSCI had the worst outcome. In this regard, our data are in line with common assumptions about the relationship between neurological recovery and the neurological level of injury [54].

Especially in cervically injured patients, studies have shown a potential benefit of early surgery on neurological or functional outcomes. Fehlings et al. express in their work on the Surgical Timing in Acute Spinal Cord Injury Study (STASCIS), that cervical cases have tremendously more potential for recovery than thoracic cases [8]. Grassner et al. saw improved neurological and functional outcomes in cervically injured patients operated on within 8 h after injury [23]. In this study, we estimated an optimal cutpoint at 234 min after injury in cervically injured patients, based on sensitivity over 85% and maximized specificity. The sensitivity was comparably high, at 90.9%. The specificity was 68.4% and the AUC 84.2% (Figure 3). The results of the cutpoint for thoracic and lumbar injuries were inconclusive, with an AUC of 51.6% and 54.3%, respectively, and may reflect the low number of cases included in the calculation. Dedicated studies are needed to investigate the above evidence as it could influence decision making in the treatment of cervical TSCI in both surgical departments and intensive care units.

For subgroups of complete and incomplete TSCI, we detected a difference in neurological improvement. Patients with incomplete TSCI had a more favorable neurological recovery than entirely paralyzed patients. In this respect, our observation fits into the current assumptions about the regenerative capacity of incomplete TSCI [10,22,55]. Interestingly, cervical and lumbar cases contained almost equal amounts of incomplete TSCI (60.0% vs. 58.3%).

Differences within subgroups were observed and might lead to specific treatment recommendations, underlining the persistent need for more detailed subgroup analysis in TSCI regarding surgery timing.

### 4.2. Defining “Early” Surgery

The existing differences in the definition of “early” surgery, varying between hours and days, still lead to challenges in comparison between different studies. Early operative care in human studies ranged between 5 h and 4 days after the injury, whereas late care varied between 8 h and later than 5 days after the injury [55].

In 2020, Wilson et al. postulated a three-part structure of the most frequent threshold ranges: “Late” defined by 48–72 h, “early” by less than 24 h, and “ultra-early” by 8–12 h post-TSCI [22]. This classification can be viewed critically, as the word “ultra” suggests that differentiation in the 0–8 h time frame may not be necessary or even possible.

However, surgical treatment within 8 h after trauma is not only feasible in practice; there is also evidence that the chance of neurological recovery is increased [23,52].

As Ahuja et al. suggest in their 2020 review, the time from injury to decompressive surgery should be minimized due to biological rationale [56].

Therefore, we would like to suggest that the term “ultra-early” should only be used in connection with surgery within the first 4 h (<4 h). Based on the available data, we have determined the optimal cutpoint to be at 245 min (4.08 h) after injury. This would therefore be “super-early” (4–8 h). Later thresholds could be named “very early” (8–12 h), “early” (12–24 h), “intermediate” (24–48 h) and “late” (>48 h).

### 4.3. Efficacy and Feasibility of Early Surgical Treatment of the Spinal Cord

The existing prehospital and hospital logistics seem to pose barriers to early surgery (<24 h) in a significant proportion of patients, and thereby limit the possibilities to evaluate earlier timelines [22]. Samuel et al. showed in their analysis of delays in cervical spinal cord injuries that, interestingly, a large part of these delays is due to processes that occur after hospitalization [57], which is supported by our data. Nevertheless, we showed that it is quite possible to achieve aggressive timelines under certain conditions.

As MRI is usually not performed postoperatively, it is not possible to assess the efficacy of laminectomy. In addition, adequate surgical care includes decompression and stabilization, in this study referred to as “early surgical treatment of the spinal cord”. It is important to question whether and what contribution the various surgical techniques have on the course of neurological recovery.

So far, no statement can be made about the extent to which decompression alone in TSCI contributes to a change in neurological qualities [58]. All anatomical structures that may contribute to compression must be considered when decompressing [59]. Further studies are needed to gain deeper insights into the efficacy of different surgical procedures.

To improve the overall outcome after TSCI, a holistic therapy concept is needed, which takes individual aspects of the pathophysiology and neuroimmunological response into account. Thus, therapy needs to aim for minimizing neurodegeneration while strengthening neuroprotective mechanisms and promoting neuroregeneration. It seems sensible to combine different approaches under close clinical observation.

### 4.4. Limitations

We acknowledge that the power of this study is limited. Because of the low incidence of injury, patient enrollment is challenging. Although recommendations for everyday clinical practice cannot be given, this work can still be hypothesis-generating and support future argumentation. It provides an incentive to investigate whether the observed differences would occur in a well-designed, prospective multicentric study with enough power.

Due to the retrospective nature of the study, allocation bias may have occurred. Age, the severity of the injury or comorbidities may have led to a delay in surgery. Due to the prospective data collection, we are confident that we have kept this bias as low as possible.

With age, we saw significant differences between C1 and C2. This may influence our primary outcome, though evidence exists that age does not influence neurological recovery [60]. Our data confirm observations made by Ahn et al. from 2015, in which older patients waited longer for surgery [61]. In this work, the authors discussed a possible delay due to less severe injuries compared with younger patients on a basis of therapeutic bias. Wilson et al. also raised epidemiologic considerations in their work from 2016 [62]. Older patients tend to suffer low-energy trauma in the context of falls, which typically result in an incomplete cervical injury. Young patients are more likely to suffer spinal injuries in the context of high-energy work or leisure accidents. In this regard, a severe injury could trigger treatment algorithms more quickly because it is more obvious to (pre)clinical decision makers. The delay of surgery could also have arisen due to comorbidities that accumulate with increasing age. Delayed diagnosis or therapy due to a more complex patient would be conceivable.

The gradation of AIS only allows a rough differentiation of neurological injury. It may even seem inappropriate for some situations, and therefore must be interpreted carefully [34,63,64]. There is still a need for a valid outcome measure that shows subtle differences and is easy to perform. In this respect, we see potential in the determination of immunological biomarkers as well as electrophysiological parameters.

The correct calculation of the time intervals is the basis of the statistical analysis in this study. Equating the accident time with the alarm time can lead to a distorted presentation of the results, as there is no reliable way to validate the actual accident time. A quick initiation of rescue measures is, therefore, a prerequisite for a meaningful calculation of the corresponding time intervals. 

Since neurological improvement can be observed up to 12 months after the initial injury, the significance of our primary outcome is limited, as it was finally evaluated after a 3-month follow-up period.

All patients received standardized physiotherapy and ergotherapy. To what extent these influences affect the course of neurologic remission has not been conclusively determined [65]. Potentially, therapeutic bias could affect neurologic outcome. To date, it is not known how large this effect is.

## 5. Conclusions

In this study, we did not see an association between surgical treatment within the first 4 h after trauma and improved neurological recovery in TSCI. The fastest possible care for patients with SCI should always be sought. 

Different characteristics regarding the NLI subgroups were detected. For patients with spinal cord injury at the cervical level, a time frame of 4 h was associated with optimal odds of an improved primary outcome, whereas in thoracically and lumbar injured patients a cutpoint close to 4 h was favorable. Applied to clinical practice, this means that there is a time frame to transport a patient with TSCI to an appropriate hospital, which is a basic principle of the Advanced Trauma Life Support (ATLS) guidelines.

In terms of subgroups, this study supports the goal of realizing earlier timing thresholds and endorses ultra-early (<4 h) surgery after TSCI in cervically injured patients. We propose a more differentiated consideration of the time intervals within the first 12 h after injury, to better investigate issues related to this topic.

## Figures and Tables

**Table 1:** Patient demographics of C1 and C2.

	C1	C2	Total	*p*-Value
	Ultra-Early (<4 h)	Early (4–24 h)		
	(N = 46)	(N = 23)	(N = 69)	
Neurological recovery				0.112 ^a^
yes	19 (41.3%)	5 (21.7%)	24 (34.8%)	
no	27 (58.7%)	18 (78.3%)	45 (65.2%)	
Sex				1.000 ^a^
female	8 (17.4%)	4 (17.4%)	12 (17.4%)	
male	38 (82.6%)	19 (82.6%)	57 (82.6%)	
Age				0.010 ^b^
M ± SD	41.1 (15.7)	53.9 (20.2)	45.4 (18.2)	
Median (IQR)	41.0 (20.0, 86.0)	59.0 (20.0, 80.0)	43.0 (20.0, 86.0)	
Etiology of injury				0.142 ^c^
Falls	28 (60.9%)	11 (47.8%)	39 (56.5%)	
Transport activities	16 (34.8%)	10 (43.5%)	26 (37.7%)	
Sports and leisure activities	0 (0.0%)	2 (8.7%)	2 (2.9%)	
Other traumatic causes	2 (4.3%)	0 (0.0%)	2 (2.9%)	
Severity of TSCI				0.657 ^a^
Incomplete	21 (45.7%)	12 (52.2%)	33 (47.8%)	
Complete	25 (54.3%)	11 (47.8%)	36 (52.2%)	
Neurological level of injury				0.528 ^c^
Cervical	18 (39.1%)	12 (52.2%)	30 (43.5%)	
Thoracic	20 (43.5%)	7 (30.4%)	27 (39.1%)	
Lumbar	8 (17.4%)	4 (17.4%)	12 (17.4%)	
AO Classification				0.942 ^c^
A	24 (52.2%)	11 (47.8%)	35 (50.7%)	
B	8 (17.4%)	5 (21.7%)	13 (18.8%)	
C	14 (30.4%)	7 (30.4%)	21 (30.4%)	
AIS initial				0.383 ^c^
A	31 (67.4%)	13 (56.5%)	44 (63.8%)	
B	6 (13.0%)	3 (13.0%)	9 (13.0%)	
C	7 (15.2%)	3 (13.0%)	10 (14.5%)	
D	2 (4.3%)	4 (17.4%)	6 (8.7%)	
AIS final				1.000 ^c^
A	23 (50.0%)	12 (52.2%)	35 (50.7%)	
B	4 (8.7%)	1 (4.3%)	5 (7.2%)	
C	7 (15.2%)	4 (17.4%)	11 (15.9%)	
D	12 (26.1%)	6 (26.1%)	18 (26.1%)	
Time from injury to surgery (min)				<0.001 ^b^
M ± SD	191.7 (35.6)	304.9 (69.3)	229.4 (72.7)	
Median (IQR)	195.0 (118.0, 240.0)	273.0 (244.0, 463.0)	225.0 (118.0, 463.0)	
Time from injury to admission (min)				<0.001 ^b^
M ± SD	58.7 (26.1)	92.3 (51.8)	69.9 (39.7)	
Median (IQR)	53.5 (22.0, 132.0)	75.0 (46.0, 244.0)	63.0 (22.0, 244.0)	
Time from admission to surgery (min)				<0.001 ^b^
M ± SD	133.0 (36.6)	212.5 (42.5)	159.5 (53.8)	
Median (IQR)	126.5 (69.0, 210.0)	206.0 (158.0, 309.0)	158.0 (69.0, 309.0)	
Duration of surgery (min)				0.688 ^b^
M ± SD	163.8 (58.1)	169.5 (85.3)	165.7 (67.8)	
Median (IQR)	156.0 (33.0, 314.0)	141.0 (64.0, 371.0)	150.0 (33.0, 371.0)	

*Note.* Demographic and clinical characteristics of the study population. Neurological recovery was defined as improvement in AIS within 3 months after the trauma. AO, Arbeitsgemeinschaft für Osteosynthesefragen; AIS, ASIA (American Spinal Injury Association) Impairment Scale; M, Mean; SD, Standard Deviation; IQR, Interquartile Range. ^a^ Boschloo’s test. ^b^ Kruskal–Wallis test. ^c^ Fisher’s Exact Test for Count Data.

**Table 2:** Timing descriptions in NLI groups.

	Cervical	Thoracic	Lumbar	Total	*p*-Value
	(N = 30)	(N = 27)	(N = 12)	(N = 69)	
Improvement					0.101 ^a^
yes	11 (36.7%)	6 (22.2%)	7 (58.3%)	24 (34.8%)	
no	19 (63.3%)	21 (77.8%)	5 (41.7%)	45 (65.2%)	
Time from injury to surgery (min)					0.258 ^b^
M ± SD	252.1 (88.0)	212.9 (57.6)	209.9 (43.6)	229.4 (72.7)	
Median (IQR)	233.5 (125.0, 463.0)	209.0 (118.0, 385.0)	210.5 (127.0, 275.0)	225.0 (118.0, 463.0)	
Min	125	118	127	118	
Max	463	385	275	463	
Time from injury to admission (min)					0.330 ^b^
M ± SD	76.9 (51.9)	60.6 (26.4)	73.5 (25.0)	69.9 (39.7)	
Median (IQR)	65.0 (24.0, 244.0)	54.0 (22.0, 132.0)	67.5 (47.0, 118.0)	63.0 (22.0, 244.0)	
Min	24	22	47	22	
Max	244	132	118	244	
Time from admission to surgery (min)					0.088 ^b^
M ± SD	175.2 (59.2)	152.3 (48.9)	136.4 (40.4)	159.5 (53.8)	
Median (IQR)	185.5 (84.0, 286.0)	141.0 (80.0, 309.0)	150.0 (69.0, 182.0)	158.0 (69.0, 309.0)	
Min	84	80	69	69	
Max	286	309	182	309	
Duration of surgery (min)					0.124 ^b^
M ± SD	151.9 (70.7)	182.1 (64.9)	163.2 (64.0)	165.7 (67.8)	
Median (IQR)	133.0 (33.0, 314.0)	162.0 (110.0, 371.0)	152.5 (77.0, 297.0)	150.0 (33.0, 371.0)	
Min	33	110	77	33	
Max	314	371	297	371	
Severity of TSCI					0.053 ^a^
Incomplete	18 (60.0%)	8 (29.6%)	7 (58.3%)	33 (47.8%)	
Complete	12 (40.0%)	19 (70.4%)	5 (41.7%)	36 (52.2%)	
Type of plegia					<0.001 ^a^
Paraplegia	9 (30.0%)	26 (96.3%)	12 (100.0%)	47 (68.1%)	
Tetraplegia	21 (70.0%)	1 (3.7%)	0 (0.0%)	22 (31.9%)	

*Note.* The time is presented in minutes concerning the patient’s neurological level of injury (NLI). M, Mean; SD, Standard Deviation; IQR, Interquartile Range. ^a^ Fisher’s Exact Test for Count Data. ^b^ Kruskal–Wallis test.

**Table 3:** Influence of severity of TSCI on neurological improvement.

	Incomplete (N = 33)	Complete (N = 36)	Total (N = 69)	*p*-Value
Neurological improvement				0.006 ^a^
yes	17 (51.5%)	7 (19.4%)	24 (34.8%)	
no	16 (48.5%)	29 (80.6%)	45 (65.2%)	

^a^ Boschloo’s test.

## Data Availability

The datasets used and/or analyzed during the current study are available from the corresponding author upon reasonable request.

## Supplementary Materials

The following are available online at https://www.mdpi.com/article/10.3390/jcm10245977/s1, Table S1: Demographic and Clinical Characteristics of Study Population vs. Outlier, Table S2: The American Spinal Injury Association Impairment Scale (AIS).
