# Supplementary material for: Pursuing More Aggressive Timelines in the Surgical Treatment of Traumatic Spinal Cord Injury (TSCI): A Retrospective Cohort Study with Subgroup Analysis
Source: J Clin Med. 2021 Dec 20;10(24):5977. doi: 10.3390/jcm10245977 (PMC8703655; doi:10.3390/jcm10245977)
Supplement: Supplementary file 1 [file jcm-10-05977-s001.zip › jcm-1455280-supplementary.pdf]

Supplementary Table S1.

Demographic and Clinical Characteristics of Subjects.

	Study population (N=69)	Extreme Outlier (N=4)	Total (N=73)	p-value
Sex				0.180[a]
female	12 (17.4 %)	2 (50.0 %)	14 (19.2 %)	
male	57 (82.6 %)	2 (50.0 %)	59 (80.8 %)	
Age				0.297[b]
Median (IQR)	43.0 (20.0, 86.0)	58.5 (24.0, 82.0)	44.0 (20.0, 86.0)	
Severity of SCI				0.471[a]
Incomplete	33 (47.8 %)	1 (25.0 %)	34 (46.6 %)	
Complete	36 (52.2 %)	3 (75.0 %)	39 (53.4 %)	
Type of plegia				0.533[a]
Paraplegia	47 (68.1 %)	2 (50.0 %)	49 (67.1 %)	
Tetraplegia	22 (31.9 %)	2 (50.0 %)	24 (32.9 %)	
AIS initial				0.557[c]
A	44 (63.8 %)	2 (50.0 %)	46 (63.0 %)	
B	9 (13.0 %)	1 (25.0 %)	10 (13.7 %)	
C	10 (14.5 %)	1 (25.0 %)	11 (15.1 %)	
D	6 (8.7 %)	0 (0.0 %)	6 (8.2 %)	
AIS final				1.000[c]
A	35 (50.7 %)	2 (50.0 %)	37 (50.7 %)	
B	5 (7.2 %)	0 (0.0 %)	5 (6.8 %)	
C	11 (15.9 %)	1 (25.0 %)	12 (16.4 %)	
D	18 (26.1 %)	1 (25.0 %)	19 (26.0 %)	
Etiology of injury				0.436[c]
Falls	39 (56.5 %)	4 (100.0 %)	43 (58.9 %)	
Other traumatic causes	2 (2.9 %)	0 (0.0 %)	2 (2.7 %)	
Sports and leisure activities	2 (2.9 %)	0 (0.0 %)	2 (2.7 %)	
Transport activities	26 (37.7 %)	0 (0.0 %)	26 (35.6 %)	
AO-Classification				0.815[c]
A	35 (50.7 %)	3 (75.0 %)	38 (52.1 %)	
B	13 (18.8 %)	0 (0.0 %)	13 (17.8 %)	
C	21 (30.4 %)	1 (25.0 %)	22 (30.1 %)	
Neurological level of injury				0.654[c]
Cervical	30 (43.5 %)	3 (75.0 %)	33 (45.2 %)	
Thoracic	12 (17.4 %)	0 (0.0 %)	12 (16.4 %)	
Lumbar	27 (39.1 %)	1 (25.0 %)	28 (38.4 %)	

Note. This table compares clinical characteristics of the study population to excluded extreme outliers. NLI, Neurological Level of Injury, defined as the lowest neurological level, where both motor and sensory function are

intact; AO, Arbeitsgemeinschaft für Osteosynthesefragen; AIS, ASIA (American Spinal Injury Association) Impairment Scale;

[a] Boschloo's test. [b] Kruskal-Wallis test. [c] Fisher's Exact Test for Count Data.

Supplementary Table S2.

The American Spinal Injury Association Impairment Scale (AIS).

AIS Grade		Clinical State
A	Complete	No motor or sensory function is preserved in the sacral segments S4-S5
B	Incomplete	Sensory but not motor function is preserved below the NLI and includes the sacral segments S4-S5
C	Incomplete	Motor function is preserved below the NLI and more than half of key muscles below the NLI have a muscle grade less than 3
D	Incomplete	Motor function is preserved below the NLI, and at least half of key muscles below the NLI have a muscle grade of 3 or more
E	Normal	Motor and sensory function is normal

Note. This table shows AIS grades from A-E considering the completeness of paralysis and the motor and sensory function test. NLI, Neurological Level of Injury;
